# Fixed or mobile-bearing total knee arthroplasty

**DOI:** 10.1186/1749-799X-2-1

**Published:** 2007-01-05

**Authors:** Chun-Hsiung Huang, Jiann-Jong Liau, Cheng-Kung Cheng

**Affiliations:** 1 Department of Orthopaedic Surgery, Mackay Memorial Hospital, No. 92, Sec. 2, Chung-San North Road, Taipei 104, Taiwan; 2 Institute of Biomedical Engineering, National Yang Ming University, No. 155, Sec 2, Li-Nung Street, Taipei 112, Taiwan; 3 School and Graduate Institute of Physical Therapy, College of Medicine, National Taiwan University, 3F, No. 17, Xuzhou Road, Taipei 100, Taiwan

## Abstract

Fixed and mobile-bearing in total knee arthroplasty are still discussed controversially. In this article, biomechanical and clinical aspects in both fixed and mobile-bearing designs were reviewed. In biomechanical aspect, the mobile-bearing design has proved to provide less tibiofemoral contact stresses under tibiofemoral malalignment conditions. It also provides less wear rate in in-vitro simulator test. Patients with posterior stabilized mobile-bearing knees had more axial tibiofemoral rotation than patients with posterior stabilized fixed-bearing knees during gait as well as in a deep knee-bend activity. However, in clinical aspect, the mid-term or long-term survivorship of mobile-bearing knees has no superiority over that of fixed-bearing knees. The theoretical advantages for mobile-bearing design to provide a long-term durability have not been demonstrated by any outcome studies. Finally, the fixed-bearing design with all-polyethylene tibial component is suggested for relatively inactive, elder people. The mobile-bearing design is suggested for younger or higher-demand patients due to the potential for reduced polyethylene wear and more normal kinematics response after joint replacement. For younger surgeon, the fixed-bearing design is suggested due to less demand for surgical technique. For experienced surgeon, one familiar surgical protocol and instrumentation is suggested rather than implant design, either fixed-bearing or mobile-bearing.

## Background

Total knee arthroplasty (TKA) has become a standard operative procedure to relieve pain and restore function in patients with osteoarthritis or rheumatoid arthritis. Current total knee prosthesis (TKP) devices can be subdivided into two groups based on different fundamental design principals: fixed-bearing knees, where the polyethylene tibial insert locked with tibial tray, and mobile-bearing designs which facilitate movement of the insert relative to the tray [1]. In each group, the knee system can be further subdivided into three groups, posterior cruciate ligament retained, sacrificed or substituted. In mobile-bearing group, some designs allow both anterior-posterior translation and internal-external rotation at the tray-insert interface while others rotation only is facilitated at the tray-insert counterface [1].

Implant loosening and polyethylene wear in fixed-bearing knee prostheses were recognized as major causes of late failure. Fixed-bearing prosthesis with a high conformity bearing surface provides low contact stress, but produces high torque at the bone-implant interface predisposing to component loosening. Conversely, prosthesis with a low conformity bearing surface produces less constraint force that decreasing component loosening, but generates high contact stress leading to early failure of the polyethylene [2,3]. Furthermore, the kinematic conflict between low-stress articulations and free rotation cannot be solved by any fixed-bearing knee design [4].

Mobile-bearing knee prosthesis was introduced with the aim to reduce polyethylene wear and component loosening [5]. The mobile-bearing design provides both congruity and mobility in the tibiofemoral bearing surface. This allows low contact stress and low constraint force to improve wear resistance and, theoretically, to minimize loosening [4]. In addition, the mobile-bearing knee also solves the kinematic conflict of fixed-bearing knee because a high conforming articular surface can now coexist with free rotation [4].

Although mobile-bearing design has hypothetical advantages over fixed-bearing knee, both designs show excellent survival rates of up to 95% in 10-year follow-up [6-11] and comparative studies could not demonstrate the superiority of one or the other design [10,12-14]. It appears we are at a crossroad. In this article, two aspects including biomechanical and clinical analysis in both fixed and mobile-bearing designs were reviewed.

## Biomechanical review

### Tibiofemoral contact stress under malalignment conditions

**Figure 1:**
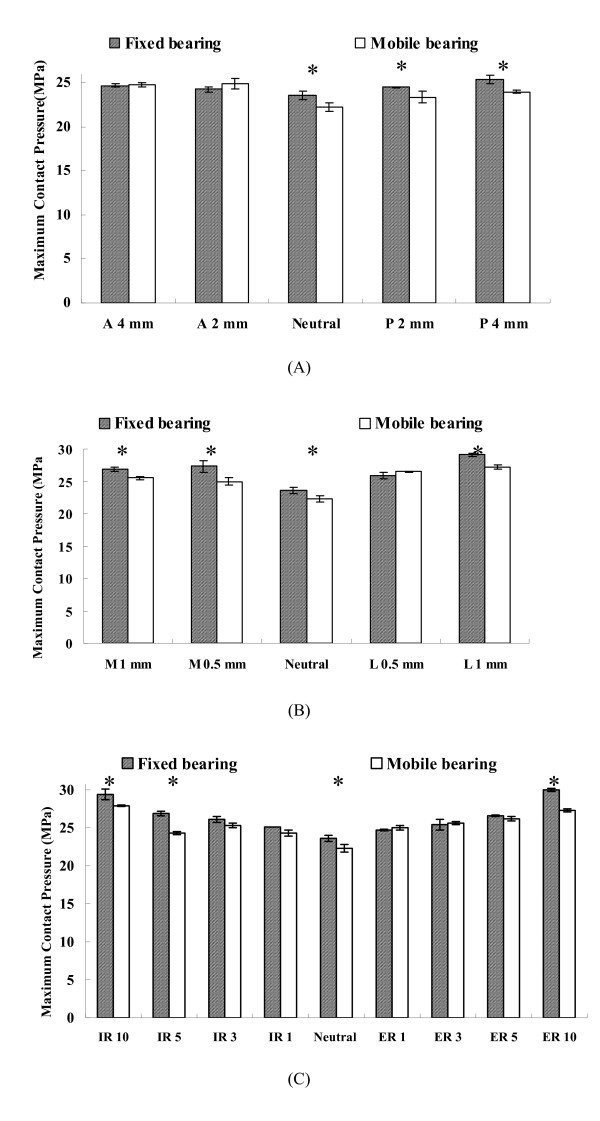
(A) Maximum contact pressures in the anterior (A) and posterior (P) maltranlations (mm) of femoral component relative to the neutral contact alignment at 0° of flexion; (B) Maximum contact pressures in the medial (M) and lateral (L) maltranlations (mm) of femoral component relative to the neutral contact alignment at 0° of flexion; (C) Maximum contact pressures in the internal malrotation (IR) and external malrotation (ER) (degrees) of femoral component relative to the neutral contact alignment at 0° of flexion. (*) indicates there is statistically difference between fixed-bearing and mobile-bearing design.

Many biomechanical studies have demonstrated that contact stress on the tibial polyethylene component is closely related to polyethylene wear [2,15]. Matsuda et al [16] measured the contact pressures on the upper- and under-surface of the tibial polyethylene insert with a neutral and malrotated tibial tray of three mobile-bearing designs and one fixed-bearing design. They found mobile-bearing design appears to offer advantages over the fixed-bearing design when moderate rotational malalignment of the tibial component occurs. Stukenborg-Colsman et al [17] used an electronic resistive pressure sensor to measure tibiofemoral contact stresses of fixed and mobile-bearing TKP under dynamic loading conditions. Stresses were measured at the tibial component aligned normally, as well as in internally and externally rotated positions. Their result showed that increase of contact stresses in a malrotated tibial component were seen with the standard conformity fixed-bearing design while these increases were not observed with the high-conformity fixed-bearing and mobile-bearing designs. They also suggested that mobile-bearing design allowing tibial insert to translate on the tibial baseplate permits the component to align itself with the femoral component so that contact area is maximized and contact stress is reduced. This feature of mobile-bearing design reduces the likelihood of cold flow and stress-peak damage. In addition, we also investigated the contact stresses in tibial polyethylene component of fixed and mobile-bearing knee prostheses under medial-lateral, anterior-posterior maltranslations and in internal-external malrotations of tibiofemoral joint [18]. Our result showed that the mobile-bearing design can reduce maximum contact pressure more significantly than the fixed-bearing design when malalignment conditions of the tibiofemoral joint occurs, especially in the internal/external malrotation (Fig 1). The mobile-bearing design offers the advantage of self-adjustment over the fixed-bearing design to accommodate surgical malalignment. Based on above-mentioned biomechanical studies, the advantage of mobile-bearing design to decrease contact pressure on the tibial articular surface under malalignment conditions of tibiofemoral joint has been proved.

### Wear rate and wear particles in fixed and mobile-bearing designs

One major goal of mobile-bearing knee is to reduce the overall wear damage by increasing the contact area, while minimizing the constraint and encouraging nature knee motion, by allowing the polyethylene bearings to move freely on polished plates on the upper tibia [19]. McEwen et al. [1] used a physiological knee simulator to compare the wear rate of fixed-bearing and rotating platform mobile-bearing TKP. The PFC Sigma rotating platform mobile-bearing knee (Depuy, Warsaw, IN) exhibited a mean wear rate of 5.2 ± 3.8 mm^3^/million cycles when subjected to standard kinematics with force-controlled anterior-posterior translation. This showed a two-fold reduction in volumetric wear rate in comparison to the fixed-bearing PFC Sigma knees subjected to intermediate kinematics (9.8 ± 3.7 mm^3^/million cycles). The authors [1] attributed the lower wear rate in mobile-bearing design to that rotating platform mobile-bearing design redistributes the motions between the femoral-insert and tray-insert articular surfaces. Most of the rotation occurs at the tray-insert articular surface, which is simply a unidirectional rotation motion and is known to produce low wear [20,21]. In contrast, rotation of the PFC Sigma fixed-bearing knee occurs entirely at the femoral-insert articulation. The resulting multidirectional wear path at the interface increases the amount of cross shear on the polyethylene articulating surface, therefore, produces a greater polyethylene wear rate [1]. However, lower wear rate cannot represent lower risk of osteolysis in vivo because the tissue response is depended on the size distributions of wear particles. When the particle size from different types of knee prosthesis were measured, the less conforming the design, the higher the surface damage, but the larger the particle size [22]. Mobile-bearing knee may have a higher rate of production of smaller particles than fixed-bearing knee due to its larger contact area. This hypothesis was disproved by an in vitro pin-on-disk wear test [19]. In that study, the authors showed that the wear rates of knee prosthesis with large contact areas, such as mobile-bearing designs, can be much less than that of fixed-bearing designs. However, there is no disadvantage regarding particle type or size associated with the larger contact areas.

### In vitro kinematics of fixed and mobile-bearing knees

In vitro cadaveric kinematics study under controlled laboratory condition was conducted by Most et al [23]. In their study, eleven human knee specimens retrieved post-mortem were tested using a robotic system. The tibiofemoral translation and rotation of the intact and two reconstructed knees, fixed-bearing posterior stabilizing knee (LPS-Flex, Zimmer, IN) and mobile-bearing posterior stabilized knee (LPS-Mobile, Zimmer, IN), were compared. One force-moment control algorithm [24] to determine the passive path from full extension to 120° and three variations of muscle loads were simulated in that study: (1) isolated quadriceps force of 400 N; (2) combined quadriceps (400 N) and hamstrings (200 N) load; and (3) isolated hamstring force of 200 N. The kinematics of the intact and reconstructed knees under these simulated muscles loads was measured at selected flexion angles (0°, 30°, 60°, 90° and 120°). Their results indicated that for all knees posterior femoral translation occurs along the passive path and under muscle loading conditions. Furthermore, increasing flexion angle corresponded with increased internal tibial rotation. Femoral translation and tibial rotation for fixed- and mobile-bearing posterior stabilized knees were similar despite component design variations. However, both reconstructed knees only partially restored intact knee translation and rotation.

### In vivo kinematics of fixed and mobile-bearing knees

In vivo kinematics studies by using fluoroscopy have been conducted on subjects with normal knee joints and on patients who had implantation of a fixed-bearing or mobile-bearing TKP [25-30]. The most important parameters, the anterior-posterior translation and axial tibiofemoral rotation, have been determined in some studies of Low Contact Stress (LCS) posterior-cruciate-sacrificing rotating platform and posterior stabilized rotating platform mobile-bearing knees (Depuy, Warsaw, IN) [25,26]. In normal knee's kinematics as the subject performed a weight-bearing deep knee-bend, there is posterior translation (14.2 mm) of lateral femoral condyle with progressive knee flexion [31]. Contact pathways in patients who had a posterior-cruciate-retaining fixed-bearing knee showed a small amount of posterior femoral rollback (4.8 mm) in the lateral component occurred during the first 60 degrees of flexion, following by anterior femoral translation as the knee flexed from 60 to 90 degrees [25]. Patients with a rotating platform knees had minimal anteroposterior tibiofemoral translation during a deep knee-bend, with tibiofemoral contact point remaining near the middle of the articular surface of tibial component [4].

Patients who had a posterior stabilized fixed-bearing TKA routinely demonstrated posterior femoral rollback in the lateral component during knee flexion, although it was less in magnitude than that in the normal knees [31]. However, patients who had a posterior-cruciate-retaining fixed bearing knee experienced a paradoxical anterior femoral translation during a deep knee-bend [32]. On the other hand, patients managed with a posterior-cruciate-sacrificing rotating platform knee replacement had posterior femoral rollback of the lateral femoral condyle from full extension to 90 degrees of flexion, but they actually experienced anterior translation from 60 to 90 degrees of flexion [4]. Patient who had a posterior stabilized rotating platform knee replacement showed more substantial posterior femoral rollback of the lateral condyle during the deep knee-bend [4].

Axial tibiofemoral rotation after TKA is another important parameter for knee's kinematics. A multicenter analysis to determine in vivo axial tibiofemoral rotational magnitude and patterns in 1,027 knees was reported [33]. In that study, normal knees showed 16.5° and 5.7° of internal tibial rotation during a deep knee-bend and gait, respectively. Patients with a posterior stabilized mobile-bearing knee prosthesis had, on average, more rotation (2.2°) than patients with a posterior stabilized fixed-bearing knee (1.4°) during the stance-phase of gait. However, patients with posterior-cruciate-retaining fixed-bearing (2.1°) knee had less rotation than patients with a posterior-cruciate-retaining mobile-bearing knee (0.1°). In a deep knee-bend activity, patients with posterior stabilized mobile-bearing design had a mean rotation of 3.9° while patients with posterior stabilized fixed-bearing one had 3.1° of rotation. Patients with posterior-cruciate-retaining mobile-bearing knee had a mean rotation of 3.9° while patients with posterior-cruciate-retaining fixed-bearing one had 3.7° of rotation. In addition, Ranawat et al [29] also investigated the tibiofemoral rotation in mobile-bearing PCL-sacrificed knee and fixed-bearing posterior stabilized knee during deep knee-bend activity. Although their results indicated that patients with a mobile-bearing prosthesis (7.3°) experienced a statistically greater amount of axial rotation than patients with a fixed-bearing prosthesis (4.1°), it is difficult to say the mobile-bearing knee has a greater axial rotation than fixed-bearing knee in vivo due to different design concept for implants, ie, PCL sacrificed vs posterior stabilized. Based on above-mentioned studies, patients with any design of knee prostheses (fixed-bearing or mobile-bearing) had smaller femoral rollback as well axial rotation than normal knees during gait and a deep knee bend. In addition, high variability in femoral rollback and axial rotation patterns and magnitudes were seen among different TKA designs.

## Clinical review

### Osteolysis and wear particle in failed fixed-bearing and mobile-bearing knee arthroplasties

**Figure 2:**
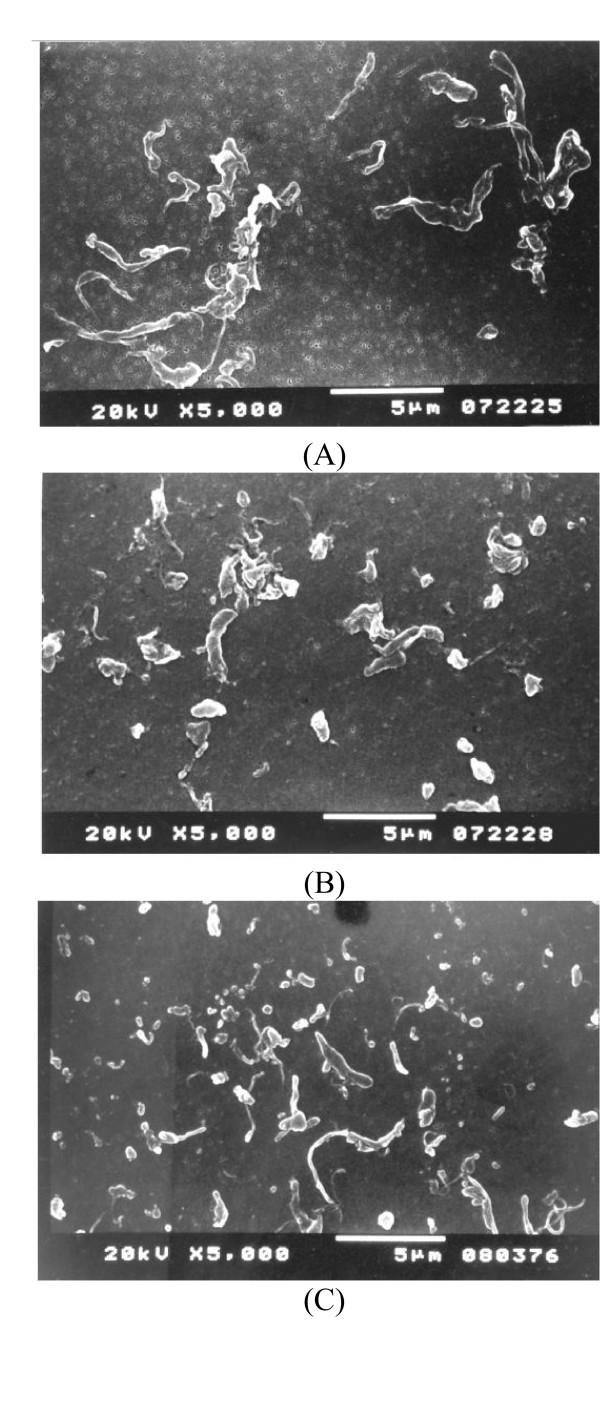
SEM microphotographs of ultra-high molecular weight polyethylene wear debris of M/G knee (A), PCA knee (B) and LCS knee (C). Note: G: granule, B: bead, F: fibril and S: larger shred.

Osteolysis has been well documented as a major complication after TKA [34-37]. The advantages of the mobile-bearing TKA are to minimise component loosening and minimise polyethylene wear. At a minimum of 15 years follow-up for cemented rotating-platform mobile-bearing TKA, Callaghan et al [38] reported no knee was revised because of loosening, osteolysis, or wear in their series of 37 patients (53 knees). However, osteolysis and polyethylene wear in rotating-platform mobile-bearing TKA was found in author's series. In our previous study [39], eighty revision TKAs with radiographic evidence of advance polyethylene wear were reviewed. The mobile-bearing group consisted of thirty-four knees (21 meniscal bearing and 13 rotating platform) with a LCS implant and the fixed-bearing group included forty-six knees. The prevalence of osteolysis was significantly higher in the mobile-bearing group (47%, 8/21 for meniscal bearing and 8/13 for rotating platform) than in the fixed-bearing group (13%). The osteolysis was predominantly on the femoral side, adjacent to the posterior aspect of the condyle. One possible reason inducing higher osteolysis rate in mobile-bearing knees was smaller phagocytosable polyethylene particles might be generated owing to the more conformed articular surface and additional undersurface wear. Although this hypothesis was disproved by an in vitro wear test [19], our another study to compare the particle size and morphology of polyethylene wear debris between failed mobile-bearing and fixed-bearing knees has proved this hypothesis [40]. In that study, tissue specimens from interfacial and lytic regions were excised during revision surgery. Ten mobile bearing knees (all of the LCS design) and 17 fixed bearing knees (10 of the porous-coated anatomic (PCA) (Howmedica, Rutherford, NJ) and 7 of the Miller/Galante design (Zimmer, Warsaw, IN) were included in this study. Polyethylene particles were isolated from the tissue specimens and examined using both scanning electron microscopy (Hitachi S-3500 N, Tokyo, Japan) and light-scattering analyses (Master-sizer 2000, Malvern, PA). The LCS mobile bearing knees produced smaller particulate debris (mean equivalent spherical diameter: 0.58 μm in LCS, 1.17 μm in PCA and 5.23 μm in M/G) and more granular debris (mean value: 93% in LCS, 77% in PCA and 15% in M/G) (Fig 2). Therefore the mobile-bearing knee may be at increased risk for osteolysis.

### Potential risks associated with mobile-bearing knees

**Figure 3:**
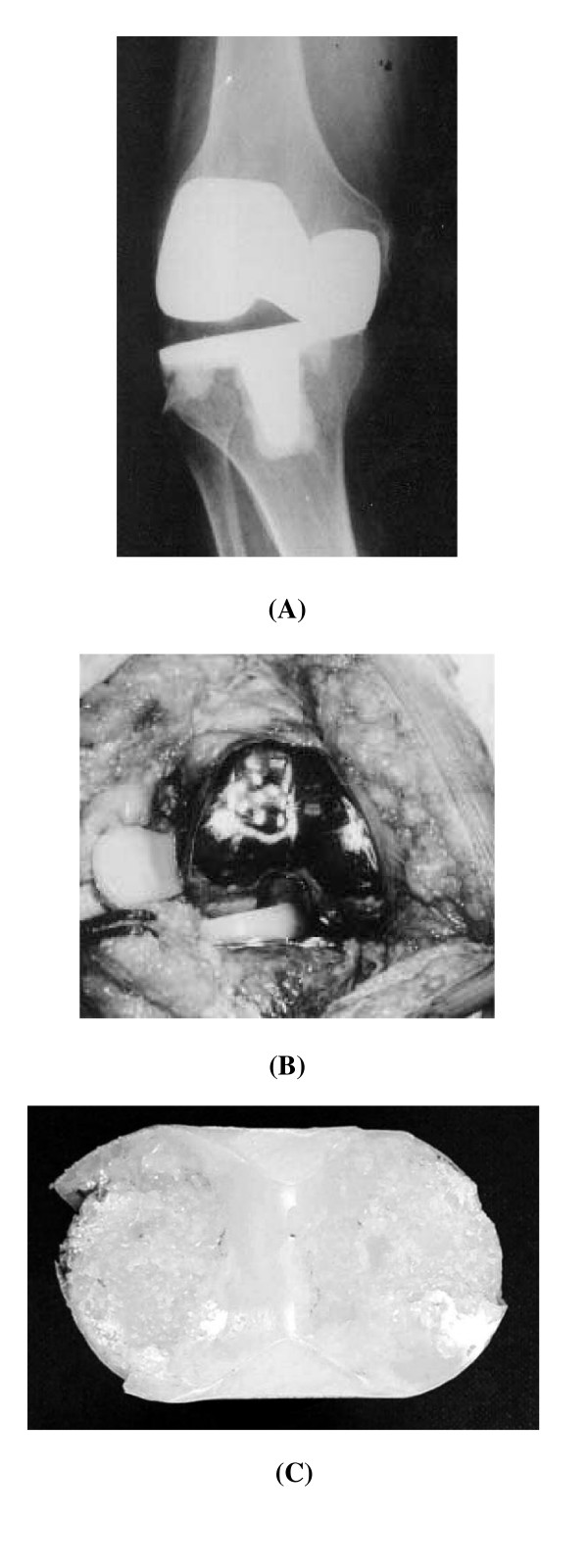
(A) An anteroposterior radiograph revealed complete absence of the femorotibial joint space. (B) At revision, the rotating platform was entrapped in the notch of the femoral component with 90° of rotation. (C) Wearing of the rotating platform is shown.

Despite many studies demonstrated advantages of using a mobile-bearing knee [4,9,38], especially for rotating platform design, several concerns have been expressed including the need for a more exacting surgical technique and the occurrence of bearing dislocation [41]. In the previous reported series [42-44], the dislocation rate in LCS knee systems occurs is less than 3.5% of cases, although Bert et al. [45] reported a higher incidence of 9.3%. All of these events occurred in early stage after knee arthroplasty and were attributed to improper surgical technique. Technical pitfalls predisposing to this complication including malrotation of tibial baseplate and failure to produce properly balanced flexion and extension tension between the femoral and tibial bearing interfaces [46]. In addition we also reported five cases with late rotational dislocation of the rotating platform bearing in the LCS knee system [41]. The prostheses had functioning well for 8 to 12 years before failure. Pre-operative radiographs showed asymmetric tibiofemoral joint spaces. Entrapment of dislocated bearing in three patients (Fig 3) and spontaneous reduction of the dislocated bearing in another two patients were seen at revision. Tibiofemoral ligamentous laxity was found after reduction. The retrieved polyethylene bearings showed advanced wear and cold flow deformities and thickness was reduced. The rotational degree of the LCS rotating platform bearing is unrestricted, which may result in late dislocation. To prevent late dislocations, a restraint mechanism is suggested to limit rotation within 30 degrees of the polyethylene element on the tibial baseplate.

### Long-term results of fixed-bearing and mobile-bearing knee arthroplasties

Some conventional fixed-bearing TKAs have been proved to be clinical successful. Survivorship of the Genesis (Smith and Nephew, Memphis, TN) TKA was 96% at 10 years follow-up [47]. Ritter et al [48] reported a survivorship of 98.8% at 15 years with the Anatomic Graduated Components (Biomet, Warsaw, IN) TKA. The survival rate of the Total Condyle knee prostheses (Howmedica, Rutherford, JN) was 95% at 15 years, 98% at 20 years and 91% at 23 years in different studies [49-51]. In our experience [52], using revision for mechanical failure as an end point, the 20-year overall survival rate with the Total Condyle knee prostheses was 91.9%. We also found the survival rate for the all-polyethylene tibial component was 96.4% and for the metal-backed tibial component was 88.4%. The use of more-cost effective and durable all-polyethylene tibial component for a primary cemented TKA, particularly in Asians with a relatively low weight and inactive, especially in elderly people is suggested.

However, in the current situation, surgeons face more and more young, active patients who need TKA. Patient's expectations for a more functional and longer-lasting result following TKAs continue to drive advances in both implant design as well as the surgical technique. The mobile-bearing knee prosthesis was designed to reduce contact stress and constraint force, which potentially, provided long-term durability. Buechel et al [8] reported a 20-year survival rate of the LCS cemented rotating platform prosthesis of 97.7% and a 16-year survival rate for the LCS cementless meniscal-bearing of 83%. Jordan et al [53] reported the survivorship of the meniscal-bearing prosthesis was 94.8% at 8 years. In our series, 495 primary LCS TKAs was reviewed [6]. Among them, 228 knees were with meniscal-bearing prostheses and the remaining 267 knees were with rotating platform. The mean follow-up was 12 years (range 10 to 15 years). The overall survivorship was 88.1% at 15 years using Kaplan-Meier analysis. The survival rate was 83% for the meniscal-bearing prostheses and 92.1% for the rotating-platform prostheses. The mobile-bearing knee prosthesis has no superiority over that of fixed-bearing knees, especially for the mensical-bearing design.

Although long-term survivorship for fixed-bearing and mobile-bearing knees have been reported, there are few studies to compare the performance of fixed-bearing and mobile-bearing in patients with bilateral TKAs. Bhan et al [54] reported their series in 32 patients who had bilateral knee arthritis with similar deformity and preoperative range of motion on both sides. Patients agreed to have one knee replaced with a mobile-bearing knee and the other with a fixed-bearing one. In a minimum follow-up of 4.5 years, the results showed that no benefit of mobile-bearing over fixed-bearing designs could be demonstrated with respect to Knee Society scores, range of motion, subjective preference or patellofemoral complication rates. The risk of bearing subluxation and dislocation in knees with the mobile-bearing prosthesis is a cause for concern and may necessitate early revision [55]. In another study which compared the mid-term follow-up of mobile-bearing and fixed-bearing in bilateral TKAs [56], the result also showed no difference between mobile-bearing and fixed-bearing prostheses. So far, the theoretical advantages for mobile-bearing design to provide a long-term durability have not been demonstrated by any outcome studies.

### Conclusion

The exact indications for using the mobile-bearing knee are still unclear. Even though the mobile-bearing design has theoretically favourable features compared with fixed-bearing system, these have not been proven biomechanically and to extend the implant longevity in clinical aspect. Based on our experience in the past thirty-year, the fixed-bearing design with all-polyethylene tibial component is suggested for relatively inactive, elder people. For younger or higher-demand patients, the mobile-bearing design is suggested due to the potential for reduced polyethylene wear and more normal kinematics response after joint replacement. For the younger surgeon, using the fixed-bearing design is suggested due to less demand in surgical technique. For the experienced surgeon, one familiar surgical protocol and instrumentation is suggested rather than implant design, either in fixed-bearing or mobile-bearing.

## Competing interests

The author(s) declare that they have no competing interests.

## Authors' contributions

CHH designed the main framework and also performed final check for this manuscript. JJL carried out the paper survey and drafted the manuscript. CKC performed the final check in this manuscript, especially in biomechanical aspect. All authors read and approved the final manuscript.
